# I Would be More at a Loss Without It: Technology as a Tool for Resilience for Older Adults During the COVID-19 Pandemic

**DOI:** 10.1093/geroni/igab046.839

**Published:** 2021-12-17

**Authors:** Rebecca Newmark, Theresa Allison, Alexander Smith, Carla Perissinotto, Ashwin Kotwal

**Affiliations:** 1 University of California, San Francisco, Oakland, California, United States; 2 University of California San Francisco, San Francisco, California, United States; 3 UCSF, UCSF, California, United States; 4 University of California San Francisco, University of California San Francisco, California, United States

## Abstract

COVID-19 associated shelter-in-place orders led to concerns about worsening social isolation and inadequate access to technology among older adults, yet little is known about technology use in this population during the pandemic. We examined older adults’ experiences with technology during shelter-in-place in order to identify lessons learned for a post-pandemic world. We conducted semi-structured in-depth interviews with a purposive sample of 20 community-dwelling older adults in San Francisco. Two independent coders conducted concurrent data analysis using inductive and deductive approaches to identify salient themes. Participants were 78 years on average (range 64-99), 55% female, 25% Black, 75% lived alone, and 60% reported at least one ADL impairment. Technology emerged as core aspect of resilience, indicating whether older adults could navigate pandemic restrictions, with two primary themes identified. First, many participants reported discovery of new technologies to maintain or develop new connections, including Zoom-based community groups and telehealth services (“there’s all kinds of virtual programs where you can exercise”). Second, older adults were resourceful in identifying community resources and enlisting family members to learn (“I had to ask one of my granddaughters how to make the chat thing work”). Despite difficulty navigating passwords, software updates and other common obstacles, most participants expressed gratitude for technology and the connectivity made possible. Many indicated an intention to integrate new technology-based social interactions into everyday life even after restrictions ended. The COVID-19 pandemic has highlighted the role technology can play in fostering resilience among older adults in adapting to external stressors.

